# Tick-Borne Encephalitis Virus Vaccines Contain Non-Structural Protein 1 Antigen and May Elicit NS1-Specific Antibody Responses in Vaccinated Individuals

**DOI:** 10.3390/vaccines8010081

**Published:** 2020-02-12

**Authors:** Jiri Salat, Kamil Mikulasek, Osmany Larralde, Petra Pokorna Formanova, Ales Chrdle, Jan Haviernik, Jana Elsterova, Dana Teislerova, Martin Palus, Ludek Eyer, Zbynek Zdrahal, Juraj Petrik, Daniel Ruzek

**Affiliations:** 1 Veterinary Research Institute, Hudcova 70, CZ-62100 Brno, Czech Republic; salat@vri.cz (J.S.); formanova@vri.cz (P.P.F.); haviernik@vri.cz (J.H.); elsterova@paru.cas.cz (J.E.); palus@paru.cas.cz (M.P.); eyer@vri.cz (L.E.); 2 Central European Institute of Technology, Masaryk University, Kamenice 753/5, CZ-62500 Brno, Czech Republic; 357352@mail.muni.cz (K.M.); zdrahal@sci.muni.cz (Z.Z.); 3 The Jack Copland Centre, Scottish National Blood Transfusion Service, 52 Research Avenue North, Edinburgh EH14 4BE, UK; olarralde-diaz@nhs.net (O.L.); juraj.petrik@nhs.net (J.P.); 4 Hospital Ceske Budejovice, B. Nemcove 585/54, 370 01 Ceske Budejovice, Czech Republic; chrdle@email.cz (A.C.); teislerova@nemcb.cz (D.T.); 5 Royal Liverpool University Hospital, Prescot St, Liverpool L7 8XP, UK; 6 Faculty of Science, Masaryk University, Kamenice 753/5, CZ-62500 Brno, Czech Republic; 7 Institute of Parasitology, Biology Centre of the Czech Academy of Sciences, Branisovska 31, CZ-37006 Ceske Budejovice, Czech Republic; 8 Faculty of Science, University of South Bohemia, Branisovska 31, CZ-37006 Ceske Budejovice, Czech Republic

**Keywords:** tick-borne encephalitis, vaccination, NS1, vaccine, flavivirus

## Abstract

Vaccination against tick-borne encephalitis (TBE) is based on the use of formalin-inactivated, culture-derived whole-virus vaccines. Immune response following vaccination is primarily directed to the viral envelope (E) protein, the major viral surface antigen. In Europe, two TBE vaccines are available in adult and pediatric formulations, namely FSME-IMMUN^®^ (Pfizer) and Encepur^®^ (GlaxoSmithKline). Herein, we analyzed the content of these vaccines using mass spectrometry (MS). The MS analysis revealed that the Encepur vaccine contains not only proteins of the whole virus particle, but also viral non-structural protein 1 (NS1). MS analysis of the FSME-IMMUN vaccine failed due to the high content of human serum albumin used as a stabilizer in the vaccine. However, the presence of NS1 in FSME-IMMUN was confirmed by immunization of mice with six doses of this vaccine, which led to a robust anti-NS1 antibody response. NS1-specific Western blot analysis also detected anti-NS1 antibodies in sera of humans who received multiple doses of either of these two vaccines; however, most vaccinees who received ≤3 doses were negative for NS1-specific antibodies. The contribution of NS1-specific antibodies to protection against TBE was demonstrated by immunization of mice with purified NS1 antigen, which led to a significant (*p* < 0.01) prolongation of the mean survival time after lethal virus challenge. This indicates that stimulation of anti-NS1 immunity by the TBE vaccines may increase their protective effect.

## 1. Introduction

Tick-borne encephalitis virus (TBEV), a member of the family Flaviviridae, genus *Flavivirus* [1], presents significant health risks in a large proportion of Europe and Asia. During the last decades, incidence rates have increased and the endemic areas have expanded [2,3]. For example, the virus has recently been detected in the United Kingdom, which was previously considered to be a TBEV-free country [4,5]. The virus is transmitted to humans by tick bites, or less frequently by consumption of unpasteurized milk or milk products from infected goats, sheep, or cows [3,6,7]. Tick-borne encephalitis (TBE) in humans can be asymptomatic or can manifest as a self-limiting, flu-like febrile disease. However, the virus can also be a cause of severe and potentially lethal neuroinfections, which most often manifest as meningitis, encephalitis, or encephalomyelitis [8]. There is no specific therapy available to treat TBE [3].

The TBEV particle is spherical, approximately 50 nm in diameter [9], and consists of three structural proteins: the capsid (C), membrane (M), and envelope (E) proteins. The viral single-stranded, positive-polarity RNA genome is bound to the C protein, forming the nucleocapsid, which is surrounded by a lipid bilayer that incorporates envelope and membrane proteins [9]. The glycosylated E protein is a major antigenic determinant of the virus and induces immune responses in infected mammalian hosts [10]. The viral RNA genome has one large open reading frame, which is translated into one large polyprotein. The polyprotein is cleaved by cellular and viral proteases into 10 proteins. In addition to the three structural proteins, seven non-structural (NS) proteins (NS1, NS2A, NS2B, NS3, NS4A, NS4B, and NS5) are produced. The NS proteins facilitate genomic replication and virion assembly. NS1 is the only viral protein secreted into the extracellular space, and is, therefore, the second main viral immunogen after the E protein [10].

TBE is preventable by vaccination, and the available vaccines are safe, and well-tolerated, and are very effective in protecting against TBE. At present, TBEV vaccines are produced commercially by five manufacturers. Two vaccines, FSME-IMMUN^®^ (Pfizer) and Encepur^®^ (GlaxoSmithKline), are manufactured in Central Europe, three in Russia, and one in China [3]. All TBEV vaccines are based on the use of formalin-inactivated, culture-derived whole virus. Vaccine-induced protection against TBEV is mediated by antibodies to the E protein. It is believed that NS1 is not present in the vaccine preparation, and therefore that vaccination alone does not give rise to anti-NS1 antibodies. This suggests that detection of anti-NS1 antibodies could distinguish antibodies induced by vaccination from those induced by infection [11] and could also considerably simplify and improve the quality of investigations of suspected TBEV infection after vaccination failures [12]. However, this study demonstrates that small amounts of NS1 can be present in TBEV vaccines and may elicit an NS1-specific antibody response in vaccinated individuals. This may increase the protective effect of TBEV vaccines, but may also complicate the distinction between serological responses following TBEV infection and vaccination.

## 2. Materials and Methods

### 2.1. Ethics Statement

The use of human patient samples was approved by the Ethics Committee of the Hospital in Ceske Budejovice (approval No. 103/19) and the Biology Center of the Czech Academy of Sciences (approval No. 1/2018).

The experiments involving laboratory animals were conducted in compliance with all relevant European Union guidelines for work with animals, and with the Czech national legal guidelines on the use of experimental animals and protection of animals against cruelty (Animal Welfare Act No. 246/1992 Coll.). The protocol was approved by the Committee on the Ethics of Animal Experimentation of the Veterinary Research Institute and the Departmental Expert Committee for the Approval of Projects of Experiments on Animals of the Ministry of Agriculture of the Czech Republic (Approval No. 14102/2015-MZE-17214).

### 2.2. Virus

The TBEV strain Hypr (a human isolate from the Czech Republic, Czech prototype strain) was used for experimental mouse infections. The virus was provided by the Collection of Arboviruses, Institute of Parasitology, Biology Center of the Czech Academy of Sciences, Ceske Budejovice, Czech Republic (http://www.arboviruscollection.cz/index.php?lang=en). Before use, the virus was passaged five times in the brains of suckling mice and in PS (porcine kidney stable) cells.

### 2.3. Vaccines

FSME-IMMUN^®^ 0.5 mL (Pfizer, New York, USA) lot numbers VNR1Q04E (MS, mass spectrometry) analysis and first vaccination experiment in mice) and VNR1S02C (second vaccination experiment in mice), and Encepur Adults^®^ (GlaxoSmithKline, Brentford, UK) lot numbers 183011A (MS analysis and first vaccination experiment in mice) and 188011C (second vaccination experiment in mice) were used in the experiments. The vaccines were stored at +4 °C before use in the experiments.

### 2.4. Mass Spectrometry

The FSME-IMMUN and Encepur Adults vaccines were processed using filter-aided sample preparation (FASP) [13]. Briefly, protein cysteine disulfide bonds were reduced in 0.1M dithiothreitol (DTT) in 8 M urea/0.1 M Tris/HCl (pH 8.5), and 50 mM iodoacetamide was used to alkylate cysteine residues. Next, proteins were digested using 500 ng of trypsin in a thermomixer at 37 °C for 4 h on a 30-kDa-filter-unit membrane and finally eluted in 50 mM ammonium bicarbonate buffer. The resulting peptides were extracted into vials with 2.5% formic acid (FA) in 50% acetonitrile (ACN) and 100% ACN with the addition of polyethylene glycol, and then concentrated in a SpeedVac concentrator.

Liquid chromatography – mass spectrometry (LC-MS/MS) analyses of peptide mixtures were done using an LCnano system (nano liquid chromatography) connected to an Orbitrap Elite hybrid spectrometer (ThermoFisher Scientific, Waltham, Massachusetts, USA). Prior to LC separation, tryptic digests were concentrated and desalted online using a trapping column (100 μm × 30 mm) filled with 3.5-μm X-Bridge BEH (ethylene bridged hybrid) 130 C18 sorbent (Waters, Milford, Massachusetts, USA). The peptides were eluted from the trapping column onto an Acclaim Pepmap100 C18 analytical column (3-µm particles, 75 μm × 500 mm; ThermoFisher Scientific) and separated using a nonlinear water/ACN (0.1% FA) gradient. The Orbitrap Elite was equipped with a Digital PicoView 550 (New Objective, Palmer, Massachusetts, USA) ion source with a sheath gas option and SilicaTip emitter. MS data were acquired using a data-dependent strategy that selected up to the top 10 precursors based on precursor abundance in the survey scan (350–2000 m/z). The resolution of the survey scan was 60,000 (400 m/z), with a target value of 1×10^6^ ions, one microscan, and a maximum injection time of 1000 ms. HCD (higher-energy collisional dissociation) MS/MS spectra were acquired with a target value of 50,000 and resolution of 15,000 (400 m/z). The maximum injection time for MS/MS was 500 ms. Dynamic exclusion was enabled for 45 s after one MS/MS spectra acquisition. The isolation window for MS/MS fragmentation was set to 2 m/z.

The mass spectrometric RAW data files were analyzed using Maxquant [14] software (version 1.6.0.13) with a built-in Andromeda search engine [15]. Data were searched against the individual sequences of the E and NS1 proteins for strain K23 (Encepur Adults) and Neudorfl (FSME-IMMUN) combined with the Maxquant contaminant database (keratin, trypsin, and others). Search settings were as follows: oxidation of methionine and deamidation of asparagine and glutamine were set as optional modifications, and carbamidomethylation of cysteine was set as a fixed modification, with two missed enzymatic cleavages considered. Only peptides and proteins with false discovery rates (FDRs; q-values) <1% were considered for final data evaluation.

### 2.5. Patient Samples

Serum samples were collected from 33 TBE patients (aged from 9 to 85 years; median, 54 years) at the acute phase of the infection. Thirty-two patients met the case definition of TBE at the time of sample collection (detection of IgM and IgG TBEV antibodies; routinely tested at the Department of Virology, Hospital Ceske Budejovice, Czech Republic). One patient was positive for TBEV IgM but negative for IgG antibodies at the time of first serum collection, but was positive for TBEV IgG antibodies at subsequent serum collections. Twenty-six serum samples were obtained from healthy blood donors who had no history of TBE or TBE vaccination. Twenty serum samples were collected from individuals (aged from 24 to 64 years; median, 31 years) vaccinated against TBE with either the FSME-IMMUN or Encepur Adults vaccine. Of these, 6 individuals received ≤3 doses of vaccine and 16 individuals received >3 doses of vaccine. All samples were investigated anonymously under random internal codes.

### 2.6. Enzyme-Linked Immunosorbent Assay (ELISA)

A TBE virus Vienna IgG ELISA kit (Euroimmun) was used to detect anti-TBEV IgG in human serum samples. The assay was performed according to the manufacturer’s instructions and the concentration of specific IgG antibodies against TBEV was expressed in Vienna units (VIEU/mL).

An Immunozym FSME IgG All Species kit (Progen GmbH) was used to detect anti-TBEV whole-virus antibodies in patient and mouse sera following the manufacturer’s instructions. This ELISA evaluates the concentration of specific IgG antibodies against TBEV in Vienna units (VIEU/mL).

A Mouse Anti-Tick-Borne Encephalitis virus NS1 IgG ELISA Kit (Alpha Diagnostic International) was used to detect anti-NS1-specific antibodies in tested sera following the manufacturer’s instructions. Concentrations of NS1-specific antibodies were expressed in arbitrary units.

### 2.7. Western Blot

Recombinant TBEV NS1 antigen produced in human 293 cells (The Native Antigen Company) was diluted to 20 ng/µL. Protein samples were prepared according to the manufacturer’s instructions for Bolt Mini Gels (Thermo Scientific). Antigen (200 ng) was loaded into each lane of a 4–12% Bis-Tris Plus Mini Gel. After electrophoresis, proteins were transferred to nitrocellulose (NC) membranes using a Trans-Blot Turbo Blotting System (Bio-Rad). NC membranes were washed once in phosphate buffered saline (PBS) with 0.05% Tween 20 (PBS-T) and blocked with 5% skim milk in PBS-T for 1 h at room temperature (RT) on a shaking platform. NC membranes were cut and incubated for 2 h at RT with human sera diluted 1:500 in 5% BSA in PBS-T. After 4 washes with PBS-T, the membranes were incubated for 1 h at RT with anti-human/HRP (Sigma) diluted 1:10 000 in 5% BSA in PBS-T. After 6 washes with PBS-T, the membranes were developed using ECL Plus Western Blotting Substrate (Pierce), and Western blot images were detected with a BioSpectrum Imaging System (UVP, Upland, USA).

The mean densities of sample and control bands on Western blots (the average of intensities of all pixels of the expected band region, minus the average intensity of background pixels) were calculated using the UVP software. The mean density of each sample band as a percentage of the positive control was calculated in Excel. Criteria for sample classification were the following: *negative*, the mean density of the sample was less than 1% of the positive control density; *low positive*, the mean density of the sample was 1–20% of the positive control density; *positive*, the mean density of the sample was more than 20% of the positive control density.

### 2.8. Immunization of Mice with TBEV Vaccines

BALB/c mice (females, 6 weeks old, Envigo; *n* = 3 to 5 per group in two independent experiments) were vaccinated subcutaneously with FSME-IMMUN or Encepur vaccine. A single immunization dose was composed of a mixture of vaccination antigen (0.25 µg), Alhydrogel adjuvant (InvivoGen, Toulouse, France) to 10% v/v, and PBS. The final volume of a single vaccine dose was 0.15 mL. Negative controls received adjuvant only; positive controls were immunized with TBEV NS1 recombinant antigen, as described below. A dose of vaccine antigens was administered every 2 weeks, for a total of six doses. Samples of blood were taken from the tail veins of mice 7 days after the third and the sixth vaccine administrations, and the concentrations of specific anti-TBEV antibodies and NS1-specific antibodies were measured by ELISA as described above.

### 2.9. NS1 Immunization in Mice

BALB/c mice (females, 6 weeks old, Envigo; *n* = 5 mice per group) were subcutaneously immunized with TBEV NS1 recombinant antigen (The Native Antigens Company). A single immunization dose was composed of a mixture of NS1 antigen (10 µg), Alhydrogel adjuvant (InvivoGen) 30% v/v, and PBS. The final volume of the single vaccine dose was 0.15 mL. A dose of NS1 antigen was administered at 2-week intervals for a total of three doses. Controls received adjuvant only. Samples of blood were taken from the tail veins of mice 7 days after the third antigen administration, and the concentration of specific anti-TBEV NS1 antibodies was measured using ELISA, as described above. To evaluate the protective effect of specific anti-TBEV NS1 antibodies, all immunized and control mice were infected intraperitoneally with a lethal dose of TBEV (10^3^ plaque-forming units (PFU) per mouse) 14 days after the third dose injection. The morbidity and mortality of the infected mice were evaluated daily throughout the period of the experiment. Mice were euthanized when serious signs of TBE neuroinfection appeared.

### 2.10. Statistical Analysis

Data are expressed as means ± standard deviation (SDs), and the significance of differences between groups was evaluated using the Mann–Whitney *U* test. To analyze categorical data, a χ2-test was used. Survival rates were analyzed using the log rank Mantel–Cox test. All tests were performed using GraphPad Prism 7.04 (GraphPad Software, Inc., Playa La Jolla, USA). A *p* value of <0.05 was considered significant.

## 3. Results

### 3.1. Mass Spectrometry Analysis

**Figure 1:**
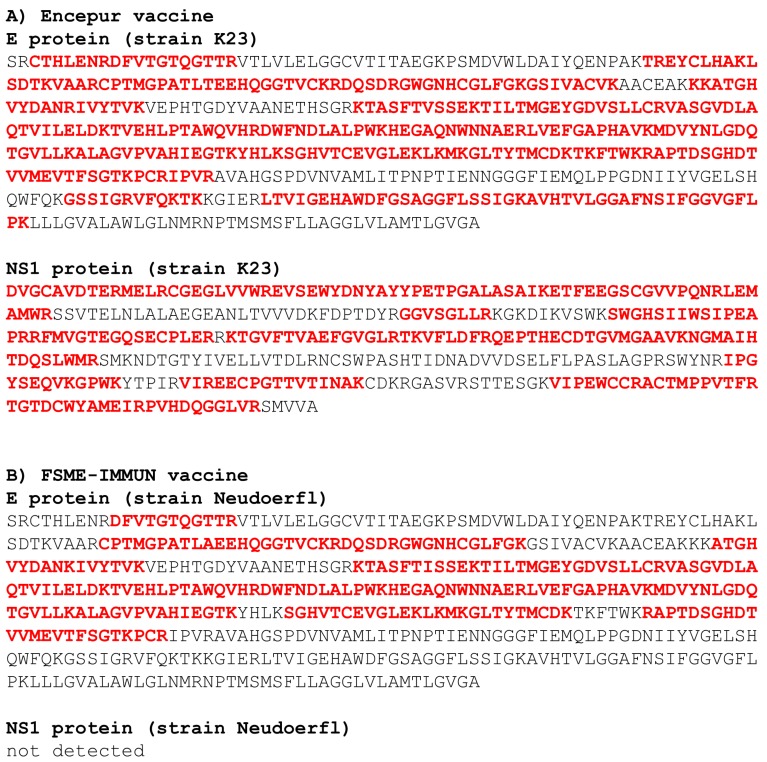
Sequence coverages of the viral envelope (E) protein and non-structural protein 1 (NS1) identified in vaccines Encepur (**A**) and FSME-IMMUN (**B**). Sequence regions identified by liquid chromatography – mass spectrometry (LC-MS/MS) are shown in bold red.

The FSME-IMMUN and Encepur Adults vaccines were processed using the FASP method [13]. The respective tryptic peptide mixtures were then subjected to LC-MS/MS analysis. Subsequent data evaluation confirmed the presence of both the E and NS1 proteins (strain K23) in the Encepur Adults vaccine. Overall, 40 and 25 peptide sequences were identified for the E and NS1 proteins, respectively. The peptides represented 68.3% and 65.6% protein sequence coverage of E and NS1, respectively (Figure 1). Based on signal intensities, the E protein was approximately 10-fold more abundant than the NS1 protein.

In contrast, only the E protein was detected in the FSME-IMMUN sample. The E protein sequence coverage was 47%, based on 24 identified peptides (Figure 1). The NS1 protein was not detected but its presence might have been obscured by the high surplus of albumin (in combination with its own lower abundance).

### 3.2. NS1-Specific Antibody Response in the Immunized Mice

**Figure 2:**
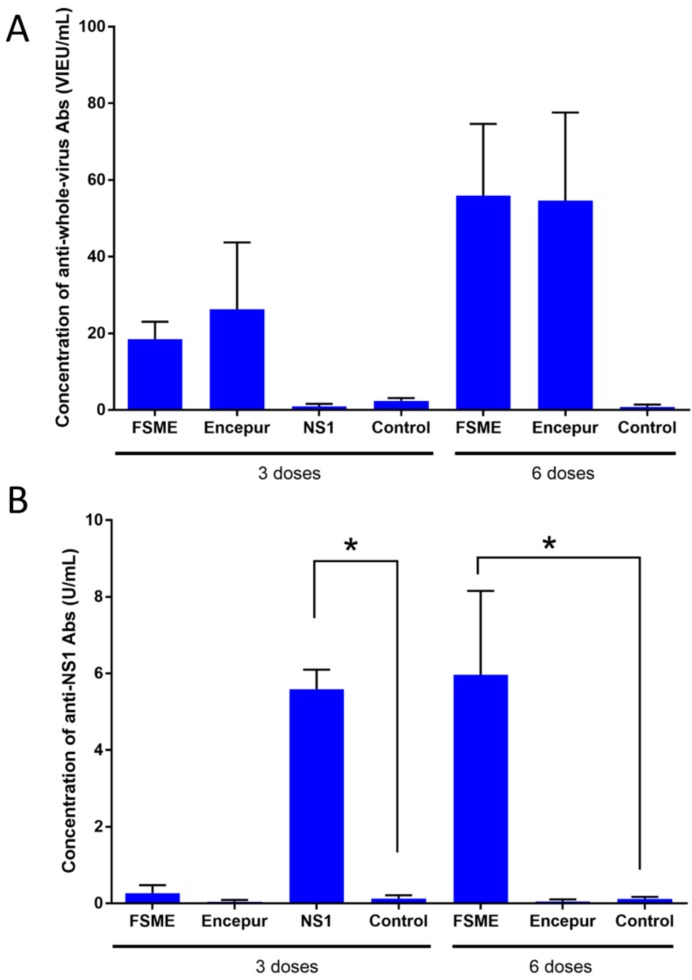
Detection of whole-virus- and NS1-specific antibodies in sera of mice immunized with tick-borne encephalitis virus (TBEV) vaccines. BALB/c mice were vaccinated subcutaneously with the FSME-IMMUN or Encepur vaccine. Negative controls received adjuvant only; positive controls were immunized with TBEV NS1 recombinant antigen (3 doses). Six doses of the vaccines were administered at 2-week intervals. Samples of blood were taken from the tail veins of mice 7 days after the third and the sixth vaccine administrations, and the concentrations of specific anti-TBEV antibodies (**A**) and NS1-specific antibodies (**B**) were measured using Enzyme-Linked Immunosorbent Assay (ELISA). Data are from two independent experiments performed with two different lots of each vaccine. *p < 0.05.

To determine whether vaccination with TBEV vaccines elicits an NS1-specific antibody response, mice were immunized with the commercially available FSME-IMMUN and Encepur vaccines. In total, the mice received six doses of the vaccine, with 2-week intervals between single doses. Negative controls received six doses of the adjuvant only. After the third and sixth immunizations, serum samples were collected and the concentrations of the anti-whole-virus and anti-NS1 antibodies were measured by ELISA. Immunization of mice with the FSME-IMMUN and Encepur vaccines elicited a robust anti-whole-virus antibody response (>20 VIEU/mL at both investigated intervals). No anti-whole-virus antibodies were detected in sera from mice that received adjuvant only or from mice that were immunized with recombinant NS1 antigen (Figure 2A). NS1-specific ELISA revealed a robust anti-NS1 antibody response in mice vaccinated with six doses of FSME-IMMUN vaccine (5.96 ± 2.19 U/mL; *p* < 0.05 compared to controls). Similar levels of NS1-specific antibodies were detected in sera of mice that were used as a positive control and received three immunizations with recombinant NS1 antigen (5.59 ± 0.51 U/mL). No NS-1 specific antibodies were detected in sera of mice vaccinated with the Encepur vaccine or in control animals that received adjuvant only (Figure 2B).

### 3.3. Detection of NS1-Specific Antibodies in Sera From TBE Patients and Vaccinees

**Figure 3:**
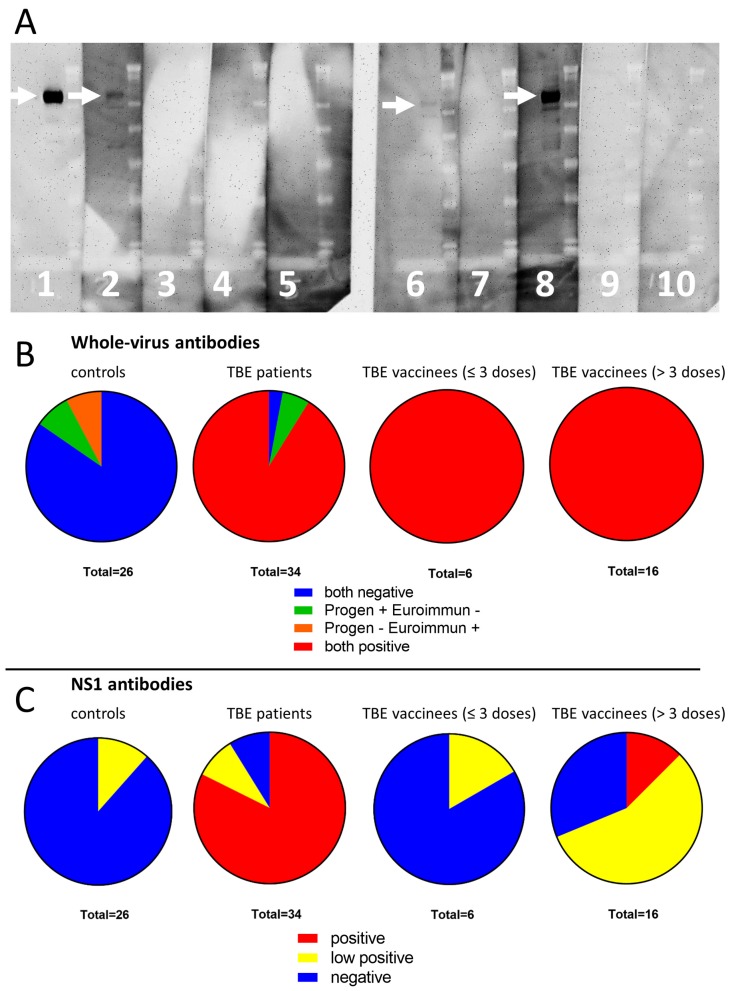
Detection of NS1-specific antibodies in serum samples of tick-borne encephalitis (TBE) patients and vaccinees. (**A**) The mean density of samples and controls (the average of intensities of all pixels of the expected band region, minus the average intensity of background pixels) was calculated and the samples were classified as follows: *low Positive*: mean density of the sample is 1–20% of the positive control mean density; *positive*: mean density of the sample is above 20% of the positive control mean density. Line 1 shows a positive control; line 8 shows a positive sample; lines 2 and 6 show examples of low-positive samples; lines 3, 4, 5, 7, and 9 represent negative samples, and line 10 shows a negative control. (**B**) Overview of the detection of TBEV-reactive IgG antibodies in all samples using two commercial ELISA assays (Euroimmun and Progen). The antibodies were detected in samples from healthy controls, TBE patients, and TBE vaccinees. (**C**) Overview of the NS1-positive, low-positive, and negative serum samples in all analyzed groups. The NS1-specific antibodies were detected in samples from healthy controls, TBE patients, and TBE vaccinees using Western blot analysis.

Serum samples from 26 healthy blood donors, 34 patients with acute TBE, and 22 TBE vaccinees were analyzed for TBEV-reactive IgG using two commercial ELISA assays (Euroimmun and Progen). No serum sample from the healthy blood donors was double positive in both assays. Two samples were positive in Euroimmun but negative in Progen, and two samples were positive in Progen but negative in the Euroimmun assay. Serum samples from 31 TBE patients (91%) were positive for TBEV-reactive antibodies in both assays, two samples were Progen positive but Euroimmun negative, and one sample was negative in both assays. All serum samples from TBE vaccinees (100%) were positive for TBEV-specific antibodies in both assays (Figure 3B).

NS1-specific IgG antibodies were detected in the serum samples using a highly specific and sensitive Western blot analysis (Figure 3A). Based on the mean density of each sample band as a percentage of the density of the positive control, the samples were classified as negative, low positive, and positive. Sera from the healthy blood donors were NS1-specific IgG negative in most cases (88%); only three samples were low positive. On the other hand, sera from TBE patients were NS1-specific IgG positive in most cases (82%), with only three samples being negative and three low positive (*p* < 0.0001 compared with healthy blood donors). Of 6 serum samples obtained from vaccinees that received ≤3 doses of the vaccine, 5 were NS1-reactive IgG negative and one sample was low positive (*p* > 0.05 compared with healthy blood donors). However, in vaccinees that received >3 doses of the vaccine, the numbers of positive and low-positive samples were much higher; from 16 samples, 2 (12.5%) were NS1 IgG positive and 9 (56%) were low positive (*p* < 0.001 compared with healthy blood donors) (Figure 3C).

### 3.4. NS1 Immunization in Mice

**Figure 4:**
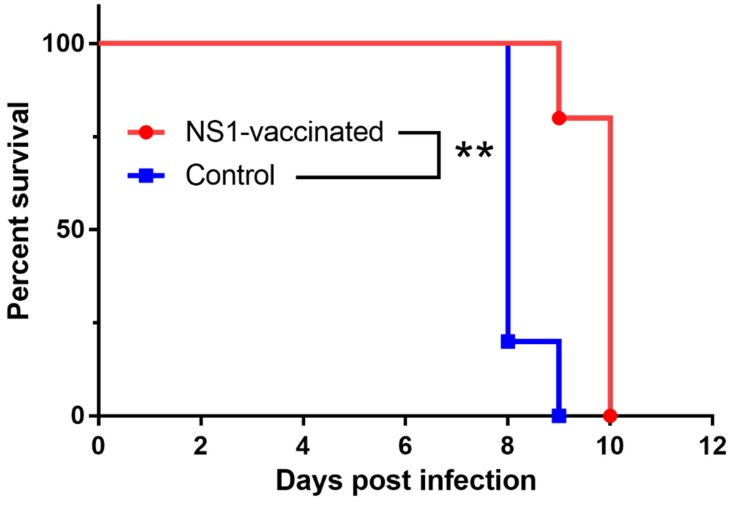
Survival of control mice and mice immunized with NS1 recombinant protein after a challenge with a lethal dose of TBEV. Immunized BALB/c mice received three doses of the recombinant NS1 antigen at 2-week intervals. Control mice received three doses of adjuvant only. On day 14 after the last immunization, all mice were inoculated with a lethal dose of TBEV and morbidity and mortality were monitored. **p < 0.01.

To determine whether NS1-specific antibodies provide protection against lethal TBEV infection, we evaluated their protective effect in our well-characterized mouse model [16,17]. Adult BALB/c mice were immunized with three doses of the recombinant NS1 antigen at 2-week intervals. The immunized animals developed a robust NS1-specific antibody response as determined by ELISA in serum samples, reaching antibody concentrations of 5.6 ± 0.5 U/mL (cut-off value = 1.0 U/mL). No NS1-specific antibodies were detected by ELISA in sera from control mice that received three doses of adjuvant only. On day 14 after the last immunization, all mice were inoculated with a lethal dose of TBEV and morbidity and mortality were monitored. The mean survival time after the virus challenge in the non-vaccinated mice was 8.2 ± 0.4 days; however, vaccinated mice exhibited a significantly (*p* < 0.01) longer mean survival time, reaching 10.2 ± 0.8 days post-infection (p.i.). Despite the differences in survival time, 100% mortality was observed in both groups (Figure 4).

## 4. Discussion

Two whole-virus formalin-inactivated TBEV vaccines (FSME-IMMUN^®^, Encepur^®^) are available in the European Union and European Economic Area [3]. Both vaccines have well-established safety profiles, and are highly effective in inducing significant immune responses in vaccinated individuals. Both vaccination and natural TBEV infection induce neutralizing antibodies directed against the main antigenic determinant of the viral particle, the E protein [18]. These antibodies are critically important in controlling and clearing the infection. However, non-neutralizing antibodies (mainly targeted against NS1) can also contribute to protection [3]. Antibodies specific for the E and NS1 antigens are usually found at high levels in the blood of infected individuals [11]. NS1 associates with intracellular and cytoplasmic membranes of the TBEV-infected cells but is also secreted to the extracellular milieu. The membrane-bound and secreted NS1 antigens are the inducers of the antibody response [19]. Since NS1 is not a structural component of the virion, it has been believed that this antigen is not present in the available vaccines based on purified and inactivated virions. Accordingly, it has been thought that detection of NS1-specific antibodies could distinguish antibodies induced by vaccination from those induced by infections [11]. Albinsson et al. [11] developed a Luminex-based TBEV suspension multiplex immunoassay, which is able to detect whole-virus and NS1 antibodies. Using this method, the authors investigated serum samples from TBE patients and TBE vaccinees with the aim of discriminating between TBE infection and vaccination, and to assess suspected TBEV vaccination failures [12]. All but two (48/50) samples from TBEV-infected patients had antibodies to NS1 antigen, as compared with only three serum samples from the vaccinees (3/50) (Albinsson et al., 2017). The authors speculated that the NS1 antibody response in these three vaccinees was due to a current or earlier TBEV infection [11]. Our study confirmed that most TBE patients have a robust NS1 antibody response; 91% (31/34) of the patients were positive or low positive for NS1-reactive antibodies in a highly sensitive Western blot analysis. However, this assay detected the presence of NS1-specific antibodies in numerous vaccinees. While those who received ≤3 doses of the vaccine were mostly NS1-antibody negative (only 1 of 6 individuals was weakly reactive), 69% (11/16) of those who received >3 doses were positive or low positive. An asymptomatic recent or previous TBEV infection in the vaccinees cannot be absolutely ruled out, however considering the control group was from the same geographic area (only 3/26 were weakly reactive for NS1 antibodies) it seems unlikely to such an extent.

These results indicate that NS1 antigen could be present in the vaccine preparations and induce the anti-NS1 immune response in the vaccinated individuals. To test the content of the vaccines, we employed mass spectrometry. The analysis of the Encepur vaccine provided clear evidence for the presence of both E protein and NS1 antigens in the vaccine preparation, corresponding to TBEV strain K23 with high sequence coverage. The mass spectrometry analysis of the FSME-IMMUN vaccine partly failed due to the high content of human serum albumin, which is used as a stabilizer in the vaccine (sucrose is used as a stabilizer in the Encepur vaccine). Thus, only E antigen corresponding to TBEV strain Neudoefl was identified in the vaccine. However, the presence of NS1 antigen in FSME-IMMUN was clearly confirmed by vaccination of naïve mice, which induced a strong anti-NS1 antibody response, in addition to anti-E antibodies. Based on these results, it can be expected that vaccination with either vaccine elicits an NS1 antibody response, and that the antibody response increases with the number of vaccine doses (boosters). The amount of NS1 antigen in the vaccine is low, and thus the antibody response to the antigen can also be relatively weak, especially when compared to NS1 antibody responses after TBEV infection. In the experimentally vaccinated mice, even 6 doses of Encepur were not sufficient to elicit a detectable anti-NS1 immune response. On the other hand, 6 doses of FSME-IMMUN elicited high levels of NS1-specific antibodies in the vaccinated animals. This indicates that the intensity of the immune response to NS1 antigen depends on the particular vaccine. In both vaccines, the seed virus is grown in primary chick embryo cells and purified after formaldehyde inactivation by continuous-flow zonal centrifugation. The infected cells secrete high quantities of NS1 antigen into the extracellular milieu, so it is not surprising that low levels of residual NS1 are also present in the purified virus stock used for vaccination. One of the possible limitations of our study is the number of analyzed vaccine lots; therefore, a variability in the NS1 presence or concentration among different vaccine lots cannot be ruled out.

The role of NS1-specific antibodies in protecting against lethal TBEV infection was evaluated in our well-characterized mouse model of the disease [16,17]. Immunization of mice with three doses of recombinant NS1 induced high levels of NS1-reactive antibodies, which significantly prolonged survival after challenge with a lethal dose of TBEV. Our results are in agreement with other studies employing TBEV NS1 as a vaccine immunogen. For example, two doses of plasmids encoding NS1 were injected intramuscularly, after which the mice were infected with the low-pathogenic TBEV strain Absettarov. The immunization provided partial protection and prolongation of survival time [20]. Partial protection and prolongation of survival was also achieved when mice were vaccinated with synthetic peptides based on structurally conserved alpha helices of TBEV NS1 [21] and when mice received heterologous prime-boost vaccination with recombinant vaccinia virus and bacterial plasmid, both carrying the TBEV NS1 gene [22,23,24], or after vaccination with replication-defective adenovirus vector expressing TBEV NS1 [25]. NS1-based subunit and DNA vaccines against diverse flaviviruses, such as Japanese encephalitis virus (JEV), dengue virus (DENV), and yellow fever virus, are currently in various stages of development [26]. Development of anti-NS1 immunity, in addition to the immune response to major structural antigens of the virus particle, provides more protection against the virus; thus, NS1 has been incorporated into multiple vaccine candidates [27]. Based on this, the anti-NS1 immunity elicited by the TBE vaccines may further increase their protective effect.

One possible mechanism of protection by NS1-specific antibodies is complement-mediated lysis of virus-infected cells following antibody recognition of cell-surface-bound NS1 [28,29,30,31,32]. However, other mechanisms could be involved, such as phagocytosis and clearance of infected cells through Fc-γ recognition of IgG2a antibodies bound to cell-surface NS1 [19,33]. In DENV infection, in addition to eliciting protective antibodies, it appears that NS1 also has the ability to induce potentially pathogenic autoantibodies [34,35,36,37,38]. However, the same effect has not been observed in JEV infection [32], indicating that the induction of autoantibodies could be dengue-specific. More research on this question with other flaviviruses, including TBEV, is needed.

## 5. Conclusions

This study provides the first evidence, to our knowledge, that TBEV vaccines FSME-IMMUN^®^ and Encepur^®^ contain NS1 antigen, in addition to the structural antigens of the whole virus particle, and that an anti-NS1 immune response can be elicited in vaccinated individuals. The induction of NS1-specific antibodies may increase the protective effect of the TBEV vaccines but may also complicate the distinction between serological responses following TBEV infection and vaccination.
